# The Duty of the Government to Its People*President’s address, fourth biennial session American International Congress on Tuberculosis, New York City, Nov. 14, 1906.

**Published:** 1906-12

**Authors:** F. E. Daniel

**Affiliations:** Austin, Texas


### TEXAS MEDICAL JOURNAL.

Established July, 1885.

F. E. DANIEL, M. D., - - - - - Editor, Publisher and Proprietor.

PUBLISHED MONTHLY.—SUBSCRIPTION $1.00 A YEAR.

VOL. XXII. AUSTIN, DECEMBER, 1906. No. 6.

The publisher is not responsible for the views of contributors.

### The Duty of the Government to Its People.*

F. E. DANIEL, M. D., AUSTIN, TEXAS.

FRIENDS AND COLLEAGUES: We meet today to consider and discuss a problem that more extensively and profoundly affects the human race than any of the questions of sociology; and upon the solution of which depend millions of lives, and the health and happiness and welfare of not only the present generation, but of thousands of unborn generations. It is our work today, to take up and further the campaign against that greatest scourge of humanity, consumption, inaugurated by the American Congress on Tuberculosis in this city six years ago, prosecuted and furthered with encouraging results at the St. Louis session of the Congress in October, 1904, and more recently by the American Anti-Tuberculosis League at Washington, and to endeavor, by calm and dispassionate discussion, to arrive at a consensus of opinion as to the proper and best course to pursue to induce our government, National and State, to apply the principles of sanitary science to the arrest of the ravages of this great scourge; to prevent the dissemination of the infection, and, as far as may be possible, to eradicate the seeds of the disease.

The importance of the subject has at last aroused world-wide attention, and it is most encouraging and gratifying to note the active interest of the Federal government and the official support given this movement; and the presence here today of scientists,

*President's address, fourth biennial session American International Congress on Tuberculosis, New York City, Nov. 14, 1906.

lay and medical, from so many and divers sections, some of whom are known to the world by their labors in the cause of humanity.

While it is expected that the discussions will take a wide range, and cover, perhaps, every phase of the problem, the keynote will be "Preventive Legislation," the application of sanitary science by authority of law, to the control of this disease; and while the problem is one of gigantic difficulties, we are encouraged by the triumphs of sanitation in recent years in the suppression and almost eradication of other deadly diseases, to hope that our labors will not be in vain. It is unnecessary to more than allude to the conquest of yellow fever, smallpox, cholera, bubonic plague, diphtheria and other diseases that once were so destructive and so dreaded,—the history of these triumphs is too well known to all present to be more than mentioned. We are, therefore, not so much concerned with the origin of the disease, and do not care whether or not it originated in Egypt and was communicated to the world by germs slumbering for centuries in the dust of mummies, as contended by Professor Sorgnac; nor with the pathology and clinical features of the disease; nor with the manner of introduction of the bacilli into the human lungs—(let it be understood we are speaking of "open consumption"—pulmonary tuberculosis)—whether they may not reach the lungs via the intestinal tract, as well as by direct inhalation of germ-laden dust; nor are we directly concerned with the treatment of the disease. Upon all these phases of the subject there is still more or less controversy and uncertainty. But we are confronted with the fact, universally recognized, that the disease is transmitted from the sick to the well by infection, and the infection is disseminated by means of germ-laden dust, principally, in homes, hotels, churches, factories, public conveyances, and in almost every place where people congregate. And while all the known or suspected methods of dissemination and entry of the infection into the human system must be considered, that they may be guarded against, yet the main question is: How may the disease be limited and controlled, and, in time, eradicated? We are confronted with the fact that the disease is endemic in every city and State in America, and in every country and clime; and that it is no respecter of persons, race, age or class; that none are immune. It is a ruthless destroyer, and may invade every home. There is no evading it. It can be contracted almost anywhere. It is the most insidious of diseases, and creeps upon one without warning, —like a thief in the night, and snatches away the brightest and best; the high and the low, the laughing babe, the radiant and loving mother, the strong man and the tottering octogenarian. It desolates homes all over this beautiful land, bringing sorrow and grief to thousands of hearts and blighting hope and paralyzing ambition; for the people believe it to be hereditary and hopelessly incurable. Like the worm in the bud, it is sapping the vitals of our race, and retarding that progress of civilization that should develop and progress *pari passu,* morally, physically and intellectually. It threatens the very integrity of our race.

In the whole range and scope of social science, as broad and comprehensive as it is, embracing, as it does, every interest of the social body of the nation,—its commercial, financial, industrial, scientific, religious, economical and political,—there is no more important subdivision than that of the public health. It is the one vital subject; the foundation upon which rest the prosperity, the integrity, the welfare, the perpetuity,—the very existence of a State or nation. It is of vastly more importance than are those conditions which affect the morals of the people, crime and prostitution, for instance, as far-reaching as are their pernicious influences; and doubtless both are influenced and morals improved by a conservation of the public health. Such a devastating disease is a factor in poverty, crime and prostitution. Legislation in the interest of the public health is legislation in the interest of public morals. The problem is of deeper importance than industrial disturbances, such as strikes, etc., or of any of the problems with which the student of sociology is confronted. And yet, strange to say, it is less studied by government, less understood, less safeguarded than any other interest, public or private, personal or property.

In a broad consideration of the subject we should take into account all those things which affect the health and threaten the life of the citizens. We must limit ourselves, however, to a brief consideration of consumption in its relation to the public, and of the best means of prophylaxis. It is a phase of the question that strikes at the underlying principle of the social fabric. It is the blight of the nation, the one chief factor in death and disease, and, perhaps, in crime, pauperism and prostitution,—the overshadowing evil that hangs perpetually like a pall upon every people. It destroys more lives than "plague, pestilence and famine, battle, murder and sudden death." It is responsible for 14 per cent of all the deaths from all causes, including war and famine and earthquakes and volcanoes. That is: about one-seventh of all those who die, die of consumption, every year, everywhere, all the time. And yet, it is a preventable disease. What a commentary upon our civilization and boasted enlightenment! We surely do hasten slowly. We make slow progress in preventive medicine, a progress out of all proportion to the advances made in every other line of human thought and activity. We have recently been put to shame by the little Japanese nation who, in their unequal struggle with Russia, were the first people on earth to practically apply the principles of sanitary science to the elimination of disease.

Few persons outside of the medical profession have an intelligent knowledge or even a conception of the extent of the devastation wrought by tuberculosis. How far-reaching and disastrous are its ravages, may be seen at a glance at a few figures of the statistician.

According to the U. S. Census Reports, there died from consumption in the United States in 1900, 111,000 persons, *so far as reported*. The registration districts in the States where records are kept and reports made, embrace only about one-third of the population; that is, the rural population is not represented in these reports. A calculation based on this fact, and on the given ratio, would give us a total for 1900 of 145,000 deaths; and for the census year 1890, 154,000, or an average, yearly, of about 150,000. Thus, in the decade between the census years there were in the United States one and one-half million deaths from consumption. In the War of Secession there was a total loss of three-fourths of a million of soldiers, all told, both armies, from all causes. It will be seen that at the present time as many persons are dying yearly from consumption as were lost yearly by both armies, from all causes, during that terrible conflict. Reed and Carroll and Agramonte are responsible for the statement (based on the statistics of yellow fever in this country from 1793 to 1900), that the total number of deaths from yellow fever was about 100,000. That is an average of 1000 a year for a century; while 150,000 die yearly of consumption; or, 50 per cent more die of consumption *every year* than perish by yellow fever in a hundred years. Or, put it thus: For every death from yellow fever there are one hundred and fifty deaths from consumption. Yet, when a case of yellow fever is discovered in one of the Southern cities, the panic created, and the frantic efforts of the authorities to suppress it, would be ludicrous, if it were not so serious. Compared with consumption, yellow fever is minor danger; yet the people become demoralized, institute shotgun quarantine, tear up railroad tracks, destroy bridges, and the authorities lock the wheels of commerce for months and months, thereby entailing losses estimated at millions of dollars. The U. S. government and the State governments are at once aroused to action, and no resource of science is neglected, and no amount of money is spared to arrest the progress of the disease. Were bubonic plague or cholera or yellow fever raging in America, and four hundred people were dying, daily, every day, all the time, year in and year out; or if a war were in progress on our soil with such a death loss, what would be the state of public sentiment? There would be a panic and a stampede. Every resource of science and skill, every influence and power and agency on the part of the government,—State, national and local,—would be invoked and put into operation to stop the fearful havoc. And yet, that is just what consumption is doing. It is killing four hundred and eleven people every day, every year, all the time; or about one person every three minutes, night and day; and no one seems to know it, or heed it, or care, except the sanitarian and the statistician, and they are powerless to prevent it.

Aside from any consideration of humanity, let us look at the question from a selfish viewpoint,—the standpoint of social economics. It is estimated that for every death from consumption there are five sick, the very large majority of whom are doomed to death. If one hundred and fifty thousand die annually, it means seven hundred and fifty thousand sick, and more or less disqualified for any kind of labor; three-quarters of a million sick and doomed to death, from one preventable disease; or, in a decade, seven and a half millions,—10 per cent of the entire population of America! It is impossible to arrive at a correct or nearly approximate estimate of the money value of these lives. Perhaps the best method of estimating the loss by sickness and death, is that employed by most statisticians: taking the incomes or earnings of the producers and wage-earners as a basis and figure it at the rate of 5 or 6 per cent interest; e. g.: one who earns $500 a year earns 5 per cent on $10,000. He is, therefore, worth $10,000 a year to the State or nation. Following out this calculation, we find that the money loss to the community from sickness and death from one cause only,—a preventable disease,—would reach figures almost incalculable, a sum more than sufficient to pay the public debt or to build an Isthmian canal every year. Professor Norton, of Yale, using the figures of Newholmes and Higgins, estimates the loss by sickness and death from all diseases, annually, at from $1,800,000,000 to $4,000,000,-

000. And Professor Lankester, before the Royal Society of England, said that it would be true economy were the British government to expend fifty million pounds annually for the protection of the public health.

Let us appeal to this phase of the subject, if the appeal to the claims of humanity does not arouse the authorities to action.

Savages and beasts must adapt themselves to their natural environment or perish; and many of the lower order of men possess this adaptability in a marked degree. But they do not readily adapt themselves to the artificial environment of civilization, and to many races our civilization is fatal. The Esquimaux sleep in an atmosphere that would stifle a civilized man; Major Gorgas, in charge of sanitary work on the Isthmus, says that the negro laborers from San Domingo sicken and die if made to live in sanitary surroundings, so long have they been adapted to the reverse; and it is said that when the missionaries put overcoats and blankets on the nude savages on an island in the South Pacific, who had been accustomed to sleep naked 'mid snow and ice, they died of pneumonia.

In this connection Dr. Senn's account of the decimation and almost extinction of the natives of the Society Islands in the Southern Pacific ocean, by the introduction, many years ago, of English civilization, illustrates the idea in a striking manner. It may be almost formulated into a rule that whisky and consumption follow the flag and the bible, as handmaids of civilization, and to some races they are as destructive as a pestilence.

Intellectual man must conquer his environment. He makes the material resources of his surroundings and the forces of nature subservient to his uses and the advancement of civilization. He seeks, finds and removes the causes of sickness and death, and makes the uninhabitable places populous and prosperous. In former years the laborers on the Isthmus of Panama perished by thousands, to such an extent that the work was marked by the skeletons of untold scores of human beings who could not "adapt" themselves to the death-dealing influences. American skill and learning have, by the application of sanitary science, robbed the swamps of the Panama Isthmus of its terrors, and soon that great engineering feat will be accomplished that shall sever the American continents, but unite the two oceans, and shorten the distance from the Occident to the Orient.

In this ability to conquer environment lies civilized man's chief claim to superiority in the animal kingdom. The inexorable law of survival of the fittest in the fierce struggle for existence, applies to races and nations as well as to individuals, and is the chief factor in their evolution into strength, stability and permanence. That nation is not the "fittest" that simply has the largest population; though population is the first requisite to national strength and expansion. A people may be so numerous, but defective in the elements of vitality that, like an overburdened fruit tree, where none of the fruit may ripen, it goes to pieces of its own weight; or they stagnate and degenerate until cholera, famine, plague or war relieves the congestion, or a stronger,—a fitter race reduces them to subjection.

"Ill fares the land to hastening ills a prey,
Where wealth accumulates and men decay."

A healthy, strong, vigorous and enlightened population makes a nation great. Hence the prime consideration is physical health. The State or nation which overlooks this, or ignores a fact so well understood that it has long since become a maxim: "The welfare of the people is the supreme law," commits a fatal error.

The death of every person, not to say adult, is a distinct money loss and a loss of potential to the State. It reduces the average longevity of the race, and affects the life expectancy.

It is a sad thing to see any one die of a preventable disease, and it is a crime chargeable against the State that any should so die. But when we see strong men, progressive and useful business men, men of bright intellects and strong arms,—upbuilders of civilization,—stricken down by a miserable typhoid or tubercle germ, from which they might and should have been protected, it is doubly sad; it is a shame, and a reproach to our government. Is it not the duty of the State, the Governors, the Congress to see that all such deaths are prevented? In the name of God and humanity, yes!

If a life is lost through neglect of some railroad company, or of the city government of some municipality, some one is responsible by law, for contributory negligence, at least, and the heirs of such person have recourse at law in suit for damages.

The Supreme Court of Texas has ruled that a railroad company is responsible, in damages, for negligence in permitting the spread of a contagious disease from their hospitals. A patient with smallpox, in his delirium, escaped from the guard and communicated the disease to a citizen. Justice Brown, in affirming the verdict of the lower court, awarding damages to the plaintiff, says: "The railroad company is liable to appellee, Wood, for damages caused to him by reason of smallpox being communicated to him and his family by Dickson (an employe) through the negligence of an agent of the railroad company." * * * "The evidence is sufficient to show liability of the appellant, and * * * the judgment is affirmed." M., K. & T. R. R. vs. Wood et al. [on appeal], S. W. Reporter, Vol. 68, p. 802.)

The railroad company enters into a contract with its employes. Each employe is taxed for the support of a hospital, and the amount of the tax is deducted from his wages. In consideration of this the company undertakes to care for all sick or wounded employes.

Every government, State and National, enters into a contract with its citizens, either written or implied. Every adult man is required to perform military duty when called on to take arms; to serve on jury; to give testimony in courts; to work on the public roads, and to pay taxes for the support of the government. In return for this he is guaranteed by law protection to his life, liberty and pursuit of happiness, and in the possession of the fruits of his labor. Every safeguard is thrown around these by special enactments by law, and no man, without warrant of law, may invade his premises or touch his person or possessions. But no protection whatever is given him in respect to his health,—the dearest interest of all,—except as against certain quarantinable diseases, and that is inadequate and unreliable. Preventable disease may enter his home, slay him and his family, and the government raises not a hand or voice to stay the execution.

The government is not subject to such suit, but it surely is morally responsible, at least, and it stands indicted before the world today for contributory negligence or unpardonable incompetency for every death from any preventable disease; and surely for the scores of lives lost in camp from preventable disease during the war with Spain. Major Seaman, Surgeon U. S. A., says that for every death in battle or from gunshot wound there were fourteen deaths in camp *at home,* among those who never reached a battlefield! Is it any wonder young men are loath to enlist in the army where the risk of death from preventable disease is fourteen times greater than in battle,—an army where such neglect or disregard of their safety is possible? Or is it surprising that on an alarm of yellow fever, remembering the government's total inability to limit its spread; remembering that at the point of invasion, either through venality, ignorance or neglect of local authorities,—generally the disease gains such headway as to be beyond control—the people having no confidence in the ability of the authorities to protect them, shut and bar the doors and guard them with loaded guns? or tear up the railroads—in their panic, isolate themselves, and whole communities,—thus subjecting some sections to threatened famine,—as has occurred more than once in the South.

That the representatives of the people's interests should so long have shut their eyes to the dangers of consumption, which are as real and as deadly, though intangible, as would be the invasion of an armed host; that they should shut their ears to the warnings of those whose profession and training peculiarly fit them for knowing and appreciating the dangers to the public health by reason of unsanitary conditions and specific infections, is as remarkable as it is disgraceful. A law to protect the public from poisonous food was antagonized and fought in the Congress of the United States seventeen years! And this apathy, this apparent indifference to the ravages of consumption—the fearful and unnecessary waste of life, is the more remarkable and is accentuated when we recall that seven million dollars are appropriated annually for the health of plants and cattle; two millions for the saving of life on the sea-coasts, and a million and a half for quarantine against yellow fever, and not a cent, so far as I am aware, is appropriated for prophylaxis of consumption.

In Texas we have a system of vital statistics for *pigs*, but none for the people, as Secretary Smith forcibly puts it. In Texas we have never been able to impress our legislators with the necessity of taking an account of population. I drew up a bill for registering the vital and mortuary statistics of the State, and in discussing it with one of the most influential Senators, he said: "I see no necessity for registering births and deaths, nor do I see how the death of babies can affect longevity." Seeing that he was about on a par, intellectually, with a child, I stated the case to him as I would have done to a child. I said: "Suppose one hundred babies are born one day, and all live to be 50 years of age; what is the average age?" He said: "Fifty years." I said: "Suppose fifty die the first year and fifty live fifty years; what is the average length of life of those hundred persons?" He said: "Fifty years."

Against such stupidity even the gods are powerless to contend.

The bill got through the Senate, but on the last round a Senator of unusual density, whose boast was that he was the watchdog of the treasury, and his watchword was "economy," got in an amendment cutting out the small appropriation necessary to make it effective, and thus the bill was killed; it is inoperative.

The registration of statistics of births and deaths is the first essential to reform in sanitation. No laws for the protection of the public health can be intelligently enacted without a knowledge of the causes and number of deaths; and it is a disgrace to the age in which we live and to our enlightened government that no such record is kept in the majority of States.

Dr. J. S. Billings says (Bulletin 15, "A Discussion of the Vital Statistics of the Twelfth Census"): "The only States which had (at the time of compilation of the twelfth census) a registration of deaths sufficiently complete to make the death rates worth calculating were: Connecticut, Maine, Massachusetts, Michigan, New Hampshire, New Jersey, New York, and Rhode Island, which, with the District of Columbia, form the group * * * of registration States." * * * "No Southern State, and no Northern State, except Michigan, had any satisfactory system of registering deaths at the time the data were collected."

[There are, however, more or less correct systems of registration in operation in many of the larger cities in the non-registration States, but the rural population is not represented.]

It should be compulsory. Every State should pass laws compelling a registration of its vital and mortuary statistics; and provision should be made for tabulating and publishing them. Without it the U. S. Census Bureau can never compile definite and reliable statistics; a large part of its work at present is therefore a little better than a more or less intelligent guess.

Every advance in science, the discovery of every truth, has to fight for recognition against blind bigotry, ignorance and prejudice.

It is truly remarkable how near we have often been to discoveries of priceless value, how many glimpses we have had of precious truths,—yet their recognition has been thwarted by unreasonable opposition,—or it has been established only after long and bitter controversy. It is now recognized that air, light and pure water are the life essentials,—the people are beginning to understand that "night air" and cold air and cold water, are not poisonous, and that the principal, if not the only chance, for the recovery of a consumptive is that he should "return to nature,"—live out of doors and sleep out of doors; and since the King of England endowed a great sanatorium for the hygienic treatment of consumption, and paid Dr. Latham $2500 for the best paper on the subject, the establishment of out-door sanitaria has become popular and successful. New York State has reason to be proud of her typical institutions, one at Saranac and another,—under military discipline,—in Sullivan county. Dr. Latham's motto was: "Give him air; he'll straightway be well." And yet, sixty-six years ago, when George Bodington (in England) insisted on the importance of a generous diet and a constant supply of pure air, and propounded the terrible "heresy" that cold is never too intense for a consumptive patient; and when, in 1855, fifteen years later, Dr. Henry MacCormack, the father of the late Sir Wm. MacCormack, published a book on somewhat similar lines, and in 1861 read a paper before the Royal Medical and Surgical Society in which he advocated what are now established principles, Bodington's book met with much bitter and fierce opposition, and eventually the disapproval of his methods became so universal that patients were driven from his sanitarium, and Bodington, I believe, was finally sent to a lunatic asylum as a crazy man. The members of the Royal Society refused to pass the usual vote of thanks to Dr. MacCormack, because they thought the paper was written by a monomaniac.

It is only by prevention that the disease can be controlled and finally exterminated, and by hygienic measures that it can be cured. The means necessary to the first are so simple and easily used,—if enforced by authority,—that they should be instituted at once. The infective principle is disseminated in the sputa and other secretions of the patient. This, if destroyed, would render the patient impotent for harm; for the disease is in no sense "contagious," but is highly infectious. The popular impression prevails very generally, unfortunately, and it is upon the authority of those who should know better, that the disease is "catching," like measles and scarlet fever. It grows out of a confusion of terms. It is remarkable that words are used as synonyms that have, often, widely different meanings. "Contagious" and "infectious," and "communicable" are generally accepted and used as meaning the same thing, and yet there is a difference in many ways between a "contagious" disease and a "communicable" disease. A disease may be communicable without being contagious, as are typhoid fever, cholera and consumption. But all these are communicable and infectious in the sense that the poison (bacilli) thrown off by the patient in the secretions and excretions may and do infect, and hence communicate the disease to well persons. I repeat, and wish to emphasize it: consumption is a communicable disease, but it is in no sense contagious; that is, it can not be "caught by contact." That is the meaning of the word. It is not possible for a sound, healthy person, coming in contact with a consumptive body, to acquire consumption.

Nevertheless, upon the authority of the Surgeon General of the Marine Hospital Service of the United States, backed by that of the United States Treasury Department, of which the Marine Hospital Service is a sub-department or bureau, the fiat has gone out that upon a construction of the public-health-laws by the Attorney General, consumption is a "contagious disease, dangerous to the public health"; and in June, 1902, the Superintendent of Immigration of the United States issued an order, based upon this decision, that all consumptive immigrants, without distinction, rich and poor, big and little, shall be excluded from the shores of the United States. Previous to this order the Board of Immigration, after having received the opinion of the health officer of the port, had some discretion; and it was possible to admit a child afflicted with consumption, accompanied by healthy parents, or a consumptive wife along with her healthy husband, for instance; but under this ruling every consumptive must be sent back. This involves the separation of parents from child, or husband from wife,—the disruption of whole families. And upon this ruling the authorities of California, a little later, turned back a distinguished judge from the South Pacific Islands, who was journeying to Europe, because he was suffering from pulmonary consumption, and refused to let him even cross the continent! Consumption is not a quarantinable disease; but the California people advise that consumptives be excluded from the State or held permanently in lazarettos. Such action would be unwise, irrational, inhuman. It would be worse than the shotgun quarantine of former years. It would create a panic in the public, and unjustly put a stigma upon thousands of good citizens and desirable immigrants, suffering from an acquired and curable disease. The consumptive is not a danger to be shunned and fled from, like the plague. He is not a leper, "unclean," to be avoided upon all occasions. He can be rendered harmless by the observance of simple and rational measures, and many will recover under proper hygienic conditions and environments. Scores and thousands of the best people of the West and Middle West were once consumptives.

And here let me say a word about the exploded yet popular fallacy, that consumption is a hereditary disease. It is not. It can not be transmitted from parent to child, but the child may, and often does, acquire it in the way that we now know that it is communicated, by infection from the expectorated matter that contains the poison (tubercle bacilli) or perhaps by nursing a consumptive mother. A predisposition to consumption may be transmitted from an invalid parent, or weakened vitality that would predispose to almost any disease; but consumption is never "inherited."

Thus, it will be seen that the spread of the disease from the sick to the well is, theoretically, at least, a simple matter, but it requires authority of law, and abundant means and efficient action to enforce it. However, these are mere details, and much that I have said is elementary knowledge with the majority of those present and is as a thrice-told tale, for which I should apologize, but for the fact that it is hoped and believed that it will reach, interest and arouse to action the general public. The people should be made to realize the danger and taught how to avoid it, and to know the deadly danger of disregarding the warnings. How can this be done? It is possible by such publications and other means, to awaken an intelligent public sentiment that will demand reform; demand that government shall protect the people from a deadly danger that they are otherwise powerless to avoid. But as to educating the masses,—that is impossible. The great majority of the struggling under-stratum of society — the class most prolific in suffering and in disseminating the disease,—are beyond the reach of lectures or literature or stereopticon illustrations or pathological exhibits. They can not be reached, or if reached, they can not be made to understand or to act. They are as children. The government must be to them *in loco parentis.* the government must think and act for them. All republican governments must be more or less paternal. Physicians and sanitarians meet and discuss these things. The discussions are published in the medical press, and exhaustive books are written, covering every phase of the subject. The recent book of Professor Huber, of this city, is such a treatise; it is an epitome of the subject, and is monumental of the present-day knowledge of tuberculosis. But the public,—even the more intelligent portion of it,—do not get the benefit of such work, and the lay press, as a rule, is very reluctant to publish such matter because, either the editors do not understand and appreciate the tremendous importance of disseminating the information, or political or other sensational matter pays better, and they "haven't room." Thus, we are like a convention of teachers holding sessions with closed doors. The pupils of the school do not get the benefit of the teaching.

The hope of ultimately exterminating the disease, however, is not so encouraging. Indeed, it is utopian. Consumption is a disease of civilization: a house disease, and is an incident of the artificial environment of life. People, in order to get artificial warmth, must shut out the cold air. They do not understand the principles or practice of ventilation and lighting. Our churches are veritable Black Holes of Calcutta, and are disease-breeders, the danger of which is little noticed or appreciated. I sat in an audience of five hundred at church one cool, crisp spring morning, where two red-hot stoves were doing their worst, and every door and window was closed, to "keep out the cold." Services lasted two hours. I estimated the capacity of the house, and by calculation ascertained that the same air had passed through the lungs of every person in the congregation every twelve minutes, or ten times during the service. Comment is unnecessary. Reform in this particular is imperative.

The problem of eradicating consumption involves a complete change in the manner of living, which is impossible. Much may be done, however, by attention to the construction of dwellings, factories, shops, railway coaches,—day and sleeping,—with reference to ventilation, heating and lighting. All upholstered furniture and equipment,—even carpets,—everything that catches and harbors germ-laden dust, should be abolished in hotels, boarding houses and sleeping cars. Dwellings and all living apartments and public conveyances should be constructed, not only for comfort and convenience, but with an eye to preserving the health,—the avoidance of the causes of disease,—which are now very generally known to medical men and must be taught to every citizen. The construction of tenement houses should be under the supervision of government officials of known honesty and capability, and the disgraceful and disastrous crowding, so prolific of disease, should be prohibited, under severe penalty. Every tenement house should be well ventilated, well lighted, properly heated in winter, and crowding of whole families into insufficient space should not be permitted. In this connection I suggest that the roofs of all such buildings should be made available as a playground for younger children by day, and for sleeping, as breathing places,—lungs,—for the occupants during the sweltering and suffocating nights of summer, when thousands are glad to be permitted to lie on the sidewalks and esteem it a privilege and a luxury to sleep on the ground or even sit out the night in the public parks.

Government out-of-doors sanatoria should be established in every State for the care of indigent consumptives,—to corral the floating consumptive population,—the great disseminators of the disease. Upon the approach of winter thousands of this class, as well as of the better class, flock to the South, returning, as a rule, upon the approach of summer. Hence, the railroads are powerful and prolific factors in the distribution of the disease; hence the necessity of strict sanitary policing and disinfection of all means of public conveyance. I believe I may claim, and I do so with satisfaction and pride, that the great State of Texas was the first to pass a law requiring this, and it applies also to all public institutions. The United States government should also establish sanatoria on a large scale for the purpose of caring for the indigent consumptive. England has set us an example that should be emulated, in the "King's Home for Consumptives," at Middlesex, Sussex, which was opened by the King in person in June last. For this great humanitarian work Sir Ernest Cassel presented the King with one million dollars. Here, also, is an example that should appeal to the numerous American millionaire philanthropists. What greater good to humanity,—what greater and more lasting monument could be built to the memory of the donor,—than the endowment of a great institution for the care of the unfortunates, who, through no fault of their own, are victims of that fell destroyer against which every citizen has a right to be protected? The name of James Smithson, who endowed the now great institution for the dissemination of knowledge, will live as long as civilization shall endure; and millions of successful and happy men will for ages rise up and call blessed the name of Stephen Girard, whose munificence made the education of indigent, friendless orphan boys possible.

This suggestion is earnestly commended to the consideration of those who would benefit their kind by a distribution of that wealth, in possession of which it is "a crime to die."

That much has been done in the direction of controlling the ravages of consumption by government, State and nation, can not be denied, and the mortality in recent years has been greatly decreased. The reforms in tenement life in this city have been marked by a gratifying improvement in the death rate. It is still enormous, being nearly 50 per cent of cases reported to your health department. Much has been done, also, in privately-owned institutions, to which I can not here allude. But the States, as a rule, have not taken action, and the efforts of the general government, if what little has been done can be called action, have been spasmodic, local and unsystematized; limited, and not sustained. I know of only two U. S. government sanitaria for consumptives. They are in New Mexico, one for the army, one for the navy,— the latter is under the management of the so-called U. S. Public Health Department and Marine Hospital Service. The United States has no public health department. There is no organization that exercises the functions of a public health department; but the Marine Hospital Service,—created originally to care for the sick marines,—has been expanded into a quarantine department, and all its labors are toward the prevention and control of epidemic diseases of foreign origin; and, like similar departments in the Southern States, the insufficient sums of money appropriated for such departments, is expended in quarantine and in fighting yellow fever and smallpox. Not a dollar, so far as I know, is expended toward the prevention of any of the many diseases that originate in our midst, and which destroy more lives than all the epidemics combined.

Organization is essential to any progress in anything. It certainly is necessary to success in combating any evil; and this we lack in our government, State and National. There should be a National Department of Public Health, in fact, as well as in name. There should be a commissioner of public health,—for surely the public health interest is as great as that of agriculture, labor or commerce,—all of which are really dependent upon it, and it is the duty of Congress to at once create such department with a minister in the President's cabinet. This is the unanimous sentiment of the medical profession of America, and of its numerous societies. It can not be too strongly emphasized. It is the first duty of the national government to create a Department of Health commensurate with the importance of the work that is peremptorily demanded by every consideration shall be done, and with the dignity of the medical profession and the greatness of this strong and progressive and enlightened nation. And in every State there should be a Department of Public Health, administered either by an expert in sanitation, or by a board of health as auxiliary to the National Health Department.

While it would be supererogation on my part to attempt to outline, even, the duties of such department, it is obvious that the first essential would be to provide for a uniform and thorough system of registration in every State, of births and deaths, with all the necessary data.

At a recent meeting of the American Association for the Advancement of Science, held at Cornell University, the question of such department was discussed, the discussion being led by Prof. J. Pease Norton, of the Department of Economics of Yale. He strongly advocated it, and said that the wastes from disease in the United States have become so great as to make a national department of health a necessity. I quote from his remarks:

"One million five hundred thousand persons must die in the United States during the next six months. Four million two hundred thousand persons will be constantly sick. At least five million homes, consisting of twenty-five million persons, will be made more or less wretched by mortality and morbidity." He says:

"Sickness is a radiating center of anxiety; and often death in the prime of life closes the gates of happiness on more than one life. Let us not forget that the 'bitter cry of the children' still goes up to heaven and that civilization must hear, until at last it heeds, the imprecations of forever wasted years of millions of lives.

"We look in horror on the Black Plague of the Middle Ages, yet it was a mere passing cloud compared to our own White Waste. It is reasonably certain that of the people living today over eight millions will die of tuberculosis, but not a hand is raised by the Federal government to save them. Over six millions must die of diseases of the heart and eight millions of pneumonia, but not a wheel of the official machinery at Washington is set in motion for their cure, and the event is accepted by the American population with as resigned a mien as the Hindoos show in awaiting the day of the cholera.

"On the other hand, the national government expends seven million dollars annually on plant health and animal health, but, save for the work of Doctors Wiley, Atwater, and Benedict, not one cent is expended directly on the health of infants. Thousands have been expended in stamping out cholera among swine, in saving the lives of elm trees, in importing Sicilian bugs to fertilize fig blossoms, in ostracizing certain species of weeds, in exterminating parasitic growths, but not one cent has been appropriated for eradicating pneumonia (or consumption) among human beings. The logic that justifies an annual appropriation of $2,000,000 for a life-saving service should justify protection against accidents of disease and death."

Could any argument be stronger? The lawmakers must heed such outspoken and unanimous sentiment. The people, the real source of power, must demand it.

The question of the prevention of disease is not strictly one for the medical man. It must be dealt with by law and lawmakers. The medical profession have done their full share of the work, and must now look to the people to see that it is completed.

I quote from my distinguished colleague, Professor Hughes, of St. Louis: "The people now know that the promiscuous tuberculosis spitter in public places may kill with his deadly saliva; they know that food and drink and second-hand clothing may spread it; that glasses at bars and soda fountains, dipped in water not often renewed, may spread it. That dairies may be as deadly as the apothecaries' poison; and likewise the butchers' stalls and the beef packing places; that the dust of the street may carry the seeds of death, and edibles, exposed for sale, unprotected from dust, may kill while they nourish. But enough of the people do not yet know or rightly ponder these fatal facts, to demand safety. The ice man still drags his ice blocks over the filthy pavements; putrifying garbage is still exposed in the alleys or carted through the streets, and the street car hog expectorates disease upon the floors; but publicity of the dangers of tubercular infection will spread through efforts like the Congress is now making, as publicity of political crime has spread in Missouri, till popular knowledge and demand for remedy will secure the people's rescue."

How much longer will an intelligent government deal with effects and ignore causes? How much longer will the people look to doctors to cure disease, instead of to authorities to prevent it? Medical science has discovered, isolated, demonstrated and written the life history of numerous disease-producing germs, and of disease-dispersing insects and parasites. They have pointed out the method of propagation and dissemination of diseases, and the methods by which they may be prevented. They have called in vain for government aid in the enforcement of such measures, and here and now the aid of the people is invoked. This Congress should be a popular movement, guided by medical and other sanitarians, and it is hoped and expected that the people will sustain it until the time shall come when the great destroyer will be, like yellow fever and smallpox, disarmed and defeated. The medical profession has been too long the people's unrecognized and unthanked providence in sanitary matters. The time has come when the people should take up the work and insist that the voice of science shall be heard and heeded; that the great truths revealed by long and laborious investigation and experimentation, truths vital to the dearest interests of mankind, shall be utilized in the saving of life.

That the general government should at once take prompt and adequate steps to arrest the fearful and needless waste of life by tuberculosis, is demanded by every consideration of humanity, economy and national interest and prosperity. It is the paramount duty of the hour.

